# Network Meta-Analysis of the Effects of Different Types of Traditional Chinese Exercises on Pulmonary Function, Endurance Capacity and Quality of Life in Patients With COPD

**DOI:** 10.3389/fmed.2022.806025

**Published:** 2022-02-02

**Authors:** Lingling Li, Hailiang Huang, Jiao Song, Ying Yu, Yuqi Jia, Yajie Wang, Xiaowen Dang, Lei Huang, Xinyue Liu

**Affiliations:** ^1^College of Rehabilitation Medicine, Shandong University of Traditional Chinese Medicine, Jinan, China; ^2^College of Health, Shandong University of Traditional Chinese Medicine, Jinan, China; ^3^Innovative Institute of Chinese Medicine and Pharmacy, Shandong University of Traditional Chinese Medicine, Jinan, China; ^4^College of Physical Education, Hebei Normal University, Shijiazhuang, China

**Keywords:** traditional Chinese exercises, chronic obstructive pulmonary disease, pulmonary function, exercise endurance, quality of life, network meta-analysis

## Abstract

**Background:**

In recent years, Chinese and international studies have reported that traditional Chinese exercises (TCEs) have good therapeutic effects on pulmonary function, endurance capacity, and quality of life in patients with chronic obstructive pulmonary disease (COPD). However, only a few studies have reported the differences in the efficacy of different TCEs in the treatment of COPD.

**Objective:**

The objective of this study is to compare the effects of five TCEs on patients with COPD, including Taijiquan (TJQ), Baduanjin (BDJ), Liuzijue (LZJ), Wuqinxi (WQX), and Yijinjing (YJJ).

**Methods:**

All randomized controlled trials (RCTs) of TCEs for patients with COPD were searched in PubMed, Web of Science, Cochrane Library, Excerpt Medica Database (EMBASE), China National Knowledge Infrastructure (CNKI), China Biology Medicine database (CBM), China Scientific Journal Database (VIP), and Wanfang database. The search period was from the establishment of each database to August 16, 2021. The quality of the included studies was assessed according to the Cochrane handbook of systematic review, and the network meta-analysis was conducted with R 4.0.2 (Ross Ihaka, Auckland, New Zealand) and ADDIS 1.16.8 (Gert vsn Valkenhoef, Groningen, Netherlands). The effect size was evaluated using the mean difference (MD) and 95% confidence interval (CI).

**Results:**

A total of 53 RCTs involving 3,924 patients were included. The network meta-analysis results showed that WQX was the most effective in improving FEV_1_/FVC% score and 6-MWT score. The difference was statistically significant (MD = 8.62, 95% CI 4.46 to 13.04, *P* < 0.05), (MD = 74.29, 95% CI 47.67 to 102.24, *P* < 0.05). However, YJJ was the most effective in reducing the CAT score, and the difference was statistically significant (MD = −8.38, 95% CI −13.24 to −3.28, *P* < 0.05).

**Conclusion:**

The existing evidence shows that WQX has advantages over other TCEs in improving pulmonary function and endurance capacity in patients with COPD, while YJJ has advantages in improving the quality of life. Although TCEs show no significant adverse effects, more large-scale, double-blind, and high-quality RCTs are needed in the future to verify the findings of this study.

**Systematic Review Registration:** https://www.crd.york.ac.uk/PROSPERO/, identifier: CRD42021293640.

## Introduction

Chronic obstructive pulmonary disease (COPD) is a respiratory system disease characterized by persistent airflow restriction, which develops gradually and is not completely reversible (1, 2). Studies have shown that the morbidity and mortality of COPD remain high all year round, making it the third leading cause of death after ischemic heart disease and stroke. Among the deaths induced by COPD, 90% occur in low-income and middle-income countries (3). Globally, the burden of COPD is expected to increase in the coming decades due to continued exposure to risk factors and the aging population (4). Nevertheless, this disease can be prevented and treated. That is to say, standardized rehabilitation for COPD patients can delay the acute exacerbation and progression of the disease, improve their quality of life, and reduce the disability and mortality rates (4).

The goal of COPD rehabilitation treatment is to improve refractory and intractable dysfunction, restore effective abdominal breathing, improve respiratory function, and increase respiratory efficiency (5). During treatment, various measures are taken to reduce and treat complications, improve pulmonary function and physical strength, and recover mobility, thus improving the quality of life, reducing hospitalization rates, and extending life expectance (5). At present, pulmonary rehabilitation is considered an important component of comprehensive care for patients with COPD. Exercise is regarded as a core element of pulmonary rehabilitation (e.g., endurance training), with the main aim of improving the aerobic capacity of patients (6). Most patients with COPD have difficulty maintaining high-intensity and sustained exercise training because of dyspnea, fatigue, and being old (6). Therefore, there is an urgent need to find a personalized exercise mode with moderate and low intensity.

The traditional Chinese exercises (TCEs) has been inherited and reformed, based on the holistic view of human life, combining Chinese medicine health care with Chinese medicine health preservation, which plays a very good role in strengthening body and preventing disease (7). In recent years, a growing number of studies have reported that TCEs can be used as an alternative therapy for pulmonary function, endurance capacity, and quality of life in patients with COPD. However, the sample sizes of single studies are small, and the inclusion criteria and methods vary from different studies. The lack of evidence-based studies on the efficacies of different TCEs in treating COPD makes it difficult to guide clinical practice.

Meta-analysis allows the comparison of the effects between two kinds of interventions. Compared with meta-analysis, the network meta-analysis has the advantage of providing a comprehensive and quantitative analysis of the relative effects of different interventions in treating the same disease. The network meta-analysis also provides a reliable, comprehensive, and robust evidence-based basis for selecting clinical treatment schemes by sorting out the advantages and disadvantages of efficacy and safety (8). The objective of this study is to compare the effects of five TCEs on COPD by network meta-analysis and probability ranking, thus providing a reference for selecting clinical treatment optimization schemes.

## Materials and Methods

This systematic review and network meta-analysis has been registered on the international system evaluation registration platform PROSPERO (NO. CRD42021293640). This study was performed according to the Cochrane Handbook of Systematic Review and followed the statement of preferred reporting item of systematic review and meta-analysis (PRISMA) for network meta-analysis (9).

### Data Sources and Search Strategy

All articles on TCEs for patients with COPD were searched by two reviewers in PubMed, Web of Science, Cochrane Library, Excerpt Medica Database (EMBASE), China National Knowledge Infrastructure (CNKI), China Biology Medicine database (CBM), China Scientific Journal Database (VIP), and Wanfang database. The search period is from the establishment of each database to August 16, 2021. A combination of Mesh words and free words search method was used in each database for comprehensive searches.

Taking PubMed database as an example, the search strategy is (traditional Chinese exercises OR traditional exercise therapy OR traditional exercise OR health qigong OR qigong OR tai chi exercise OR tai chi OR taijiquan OR liuzijue OR baduanjin OR eight section brocade OR wuqinxi OR yijinjing) AND (chronic obstructive pulmonary disease OR COPD OR chronic obstructive lung disease OR chronic obstructive airway disease OR chronic airflow obstruction).

### Inclusion and Exclusion Criteria

The inclusion criteria of this study was based on the five main principles of the Participant-Intervention-Comparator-Outcomes-Study design (PICOS).

#### Participants

Patients were diagnosed with COPD based on the 2017 Global Initiative for COPD (10) or other similar diagnostic criteria.

#### Interventions

The interventions are TCEs, including Taijiquan (TJQ), Baduanjin (BDJ), Liuzijue (LZJ), Wuqinxi (WQX), and Yijinjing (YJJ).

#### Comparator

The comparator is non-exercise intervention.

#### Outcome Indicators

The outcome indicators include the ratio of forced expiratory volume in the first second to forced vital capacity (FEV1/FVC%), the 6-min walking test (6MWT), the COPD assessment test (CAT) and adverse events.

#### Studies

The studies are randomized controlled trials (RCTs) of TCEs for patients with COPD. The language of studies is limited to Chinese and English.

#### Exclusion Criteria

The following studies should be excluded: non-RCTs; case reports; protocols; animal experiments; meeting abstracts; reviews; studies of other interventions; treatments combined with other therapeutic interventions; studies with incomplete data; studies with non-comparable or unreported baselines; studies with no results.

### Data Extraction

At first, duplicate records were removed by EndNote X9 (Thomson ResearchSoft, Connecticut, USA) software. Then, two reviewers independently read the titles and abstracts for preliminary screening, followed by a re-screening of the full text that might meet the inclusion criteria. Finally, the following data were extracted from the studies meeting the inclusion criteria: the first author; year of publication; patient age; sample size; interventions; outcome indicators; quality assessment; other information.

### Quality Assessment of Included Studies

The quality of included studies was assessed by two reviewers according to the Cochrane handbook for systematic reviews. If the assessment results were different, the studies were referred to a third reviewer for assessment. The assessment items included random sequence generation, allocation concealment, blind experiments, data integrity, selective reporting, and other biases. The quality assessment of the included studies was based on three options: high risk, low risk, and unclear (11).

### Statistical Analysis

In this study, network meta-analysis was performed using R 4.0.2 (Ross Ihaka, Auckland, New Zealand) and ADDIS 1.16.8 (Gert vsn Valkenhoef, Groningen, Netherlands) software. FEV1/FVC%, 6MWT, and CAT are continuous variables, and the effect value was expressed by mean difference (MD) and 95% confidence interval (CI). When the 95% CI did not contain 0, there was a statistical difference between the two groups. Based on the Markov Chain-Monte Carlo algorithm, the network meta-analysis and probability ranking was carried out using four chains and consistency models. The initial value was set to 0.5, the step size was 10, and the number of iterations was 50,000 times. The first 20,000 iterations were used for annealing to eliminate the influence of the initial value, and the last 30,000 iterations were used for sampling. The potential scale reduction factor (PSRF) was calculated to evaluate convergence by comparing the variance within and between chains. A PSRF close to 1 indicated good convergence of the consistency model analysis and more reliable results (12).

## Results

### Literature Selection

**Figure 1:**
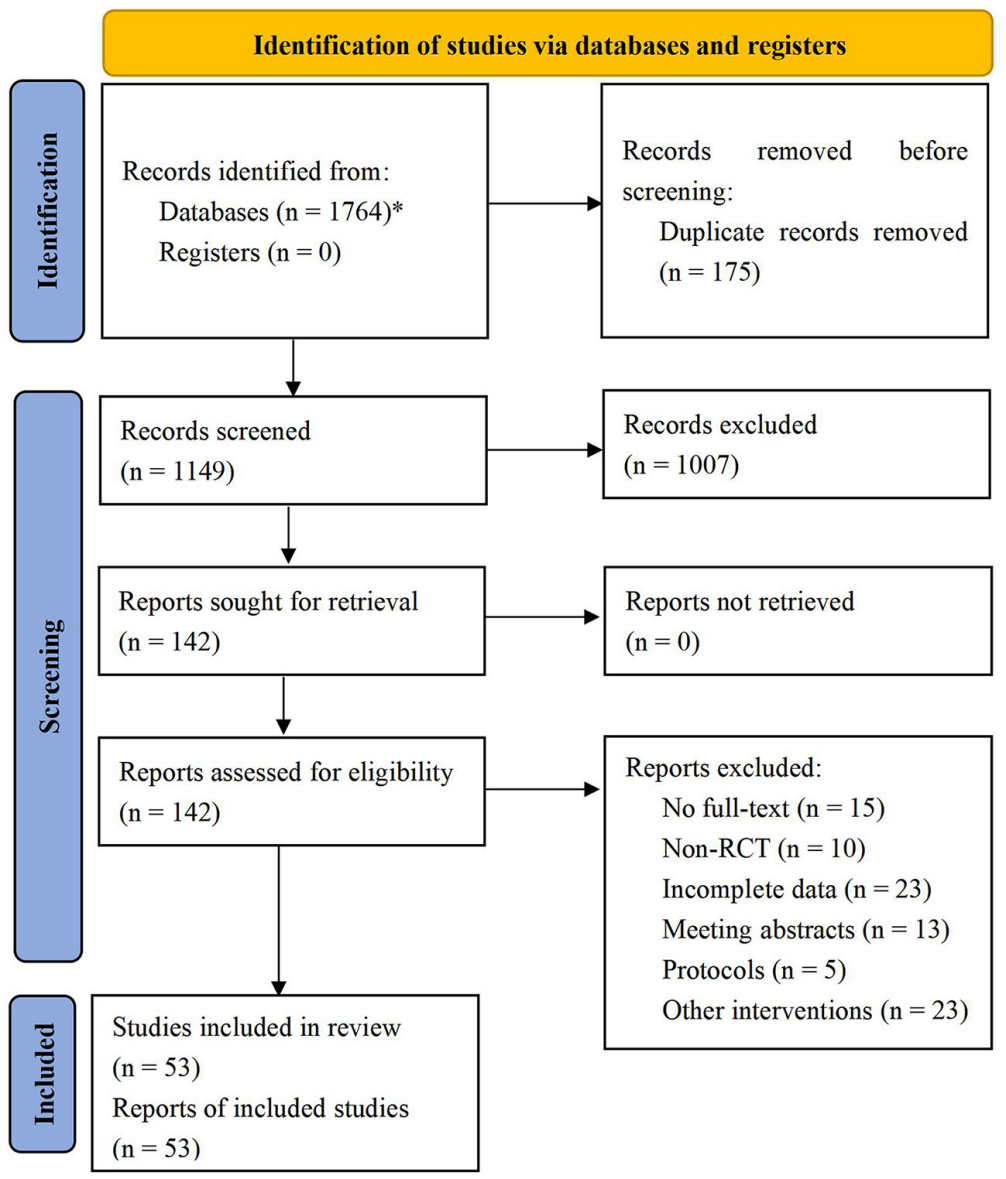
Flow diagram of literature selection. ^*^PubMed = 253, Web of Science = 247, Cochrane Library = 95, EMBASE = 104, CNKI = 271, CBM = 156, VIP = 315, Wanfang = 323.

A total of 1,764 studies were obtained from the databases, and 1,149 studies were obtained after eliminating duplicate articles. Afterward, 142 studies were selected by reading the titles and abstracts. Finally, 53 RCTs (13–65) were included by reading the full-text and excluding non-RCTs, meeting abstracts, protocols, and studies with incomplete data. The flow diagram of literature selection is shown in Figure 1.

### Characteristics of Included Studies

A total of 53 RCTs (13–65) with 3,924 patients were included in network meta-analysis, including 1,973 patients in the experimental group and 1,951 patients in the control group. The characteristics of the included studies are shown in Table 1.

**Table 1:** Characteristics of included studies.

**References**	**Simple** **Size** **E/C**	**Male/** **Female**	**Age (years)** **Mean ± SD**	**Duration of** **disease (years)**	**Intervention**		**Intervention frequency and duration**	**Outcomes**
					**E**	**C**		
Deng et al. (13)	32/32	31/1 29/3	66.26 ± 5.13/ 66.90 ± 4.63	4.68 ± 2.54/ 4.77 ± 2.52	BDJ	NEI	30 min per time, 1 time per day for 3 months	➀
Liang et al. (14)	41/41	N/A	N/A	N/A	BDJ	NEI	30 min per time, 1 time per day for 3 months	➀
Lei et al. (15)	49/50	35/14 38/12	60.45 ± 4.76/ 61.78 ± 5.32	5.37 ± 1.21/ 5.28 ± 1.12	BDJ	NEI	60 min per time, 1 time per day for 12 months	➁
Hou et al. (16)	25/23	14/11 13/10	63.34 ± 5.95/ 62.87 ± 6.01	N/A	BDJ	NEI	30 min per time, 2 times per week, for 3 months	➀➁
Wu et al. (17)	50/50	26/24 25/25	62.40 ± 5.60/ 60.70 ± 6.30	8.90 ± 3.10/ 8.40 ± 2.50	BDJ	NEI	30 min per time, 1 time per day for 6 months	➁
Guo et al. (18)	30/30	13/17 14/16	N/A	N/A	BDJ	NEI	15~20 min per time, 1 time per day for 6 months	➀
Guo et al. (19)	161/159	94/67 96/63	64.15 ± 8.97/ 64.87 ± 8.86	16.21 ± 5.53/ 16.19 ± 5.48	BDJ	NEI	30 min per time, 1 time per day for 6 months	➀➂
Deng et al. (20)	27/27	25/2 26/1	64.84 ± 9.03/ 63.92 ± 8.47	9.45 ± 3.44/ 8.56 ± 4.29	BDJ	NEI	30 min per time, 1 time per day for 6 months	➀➁
Zheng et al. (21)	19/19	18/1 18/1	69.89 ± 8.89/ 69.68 ± 6.84	N/A	BDJ	NEI	20~30 min per time, 5~7 times per week, for 3 months	➀➁➂
Sun et al. (22)	40/40	26/14 27/13	62.97 ± 6.87/ 63.21 ± 7.02	11.02 ± 3.38/ 10.85 ± 3.53	BDJ	NEI	30 min per time, 1 time per day for 12 months	➁➂
Zhu et al. (23)	63/60	36/27 31/19	69.00 ± 8.70/ 68.00 ± 9.20	12.50 ± 10.70/ 10.80 ± 8.90	BDJ	NEI	30 min per time, 2 times per day for 6 months	➀➁
Liu et al. (24)	40/40	21/19 22/18	59.77 ± 7.08/ 60.67 ± 6.95	N/A	BDJ	NEI	30 min per time, 1 time per day for 3 months	➀➁
Chen et al. (25)	39/39	74/43 73/42	60.52 ± 7.24/ 59.67 ± 6.91	N/A	BDJ	NEI	30 min per time, 1 time per day for 3 months	➀
Huang et al. (26)	31/31	20/11 18/13	68.24 ± 3.28/ 69.77 ± 4.42	2.05 ± 0.88/ 1.44 ± 1.20	BDJ	NEI	30 min per time, 2 times per day for 6 months	➀➂
Yu et al. (27)	41/41	N/A	62.30 ± 1.20/ 62.30 ± 1.50	5.30 ± 1.50/ 7.30 ± 1.40	BDJ	NEI	30 min per time, 1 time per day for 3 months	➁
Pan et al. (28)	42/42	26/16 29/13	60.70 ± 5.60/ 61.80 ± 7.20	6.70 ± 6.20/ 8.80 ± 5.30	BDJ	NEI	30 min per time, 1 time per day for 6 months	➀➁
Dong et al. (29)	46/46	27/19 25/21	63.97 ± 5.57/ 64.25 ± 6.01	N/A	BDJ	NEI	30 min per time, 1 time per day for 6 months	➁➂
Wang et al. (30)	37/36	27/10 25/11	63.17 ± 9.95/ 63.67 ± 9.75	15.17 ± 6.73/ 14.83 ± 7.89	BDJ	NEI	30 min per time, 1 time per day for 3 months	➀➁
Shi et al. (31)	20/20	18/2 15/5	71.66 ± 7.22/ 73.28 ± 6.15	N/A	LZJ	NEI	3 months	➀
Zhang et al. (32)	60/60	N/A	71.30 ± 2.96/ 72.90 ± 3.25	N/A	LZJ	NEI	30 min per time, 1 time per day for 3 months	➁
Zheng et al. (33)	30/30	13/17 16/14	N/A	N/A	LZJ	NEI	40 min per time, 5 times per week, for 6 months	➀➁
Chen et al. (34)	21/19	19/2 14/5	71.76 ± 7.31/ 73.32 ± 6.33	N/A	LZJ	NEI	3 months	➀
Peng et al. (35)	30/30	20/10 19/11	56.22 ± 4.17/ 56.17 ± 4.12	N/A	LZJ	NEI	30 min per time, 1 time per day for 3 months	➀
Hou et al. (36)	54/54	30/24 29/25	N/A	N/A	LZJ	NEI	6 months	➀
Ji et al. (37)	28/29	23/5 19/10	63.75 ± 5.48/ 64.52 ± 5.68	4.68 ± 2.61/ 3.76 ± 2.06	LZJ	NEI	30 min per time, 5 times per week, for 3 months	➀
Liu et al. (38)	27/25	14/13 13/12	66.15 ± 6.43/ 66.40 ± 8.84	9.15 ± 6.89/ 9.60 ± 6.16	LZJ	NEI	4 times per week for 6 months	➀➂
Lan et al. (39)	42/42	23/19 20/22	67.24 ± 3.21/ 67.02 ± 3.48	8.29 ± 2.76/ 8.21 ± 2.83	LZJ	NEI	5 times per week for 3 months	➀➂
Guan et al. (40)	31/32	28/3 30/2	68.52 ± 6.29/ 69.56 ± 5.13	8.19 ± 2.69/ 9.12 ± 2.08	LZJ	NEI	30 min per time, 1 time per day for 4 months	➀➁
Li et al. (41)	17/19	14/3 14/5	66.00 ± 9.00/ 66.00 ± 9.00	8.00 ± 5.00/ 9.00 ± 4.00	LZJ	NEI	60 min per time, 4 times per week, for 6 months	➀➁
Xiao et al. (42)	63/63	58/5 59/4	72.20 ± 1.70/ 70.90 ± 1.40	N/A	LZJ	NEI	30 min per time, 1 time per day for 6 months	➁
Wu et al. (43)	16/17	14/2 14/3	67.00 ± 8.00/ 66.00 ± 9.00	13.00 ± 4.00/ 12.00 ± 4.00	LZJ	NEI	40 min per time, 6 times per week, for 6 months	➀➁
Pan et al. (44)	20/21	14/6 14/7	N/A	N/A	TJQ	NEI	30 min per time, 3 times per week, for 2 months	➁➂
Zhang et al. (45)	18/18	14/4 12/6	68.02 ± 6.91/ 66.71 ± 5.84	33.41 ± 2.45/ 32.84 ± 1.98	TJQ	NEI	60 min per time, 2 times per day for 12 months	➀➁➂
Zhang et al. (46)	30/30	17/13 16/14	62.00 ± 7.30/ 62.34 ± 6.88	N/A	TJQ	NEI	1 time per day for 12 months	➀➁
Cui et al. (47)	28/28	21/7 20/8	65.80 ± 5.20/ 66.50 ± 4.80	23.50 ± 4.10/ 22.70 ± 3.60	TJQ	NEI	30~60 min per time, 2 times per day for 12 months	➀➁➂
Peng et al. (48)	40/40	16/24 18/22	N/A	N/A	TJQ	NEI	30 min per time, 1 time per day for 6 months	➀➁➂
Liu et al. (49)	50/50	30/20 28/22	53.50 ± 6.10/ 53.10 ± 5.60	9.24 ± 0.31/ 8.72 ± 0.34	TJQ	NEI	60 min per time, 1 time per day for 12 months	➀➁➂
Zhang et al. (50)	30/30	20/10 19/11	53.12 ± 6.12/ 53.62 ± 7.14	N/A	TJQ	NEI	8 months	➀
He et al. (51)	45/45	N/A	N/A	N/A	TJQ	NEI	45~60 min per time, 1 time per day for 1 month	➀➁
Yao et al. (52)	40/40	20/20 25/15	66.10 ± 4.00/ 66.20 ± 4.20	N/A	TJQ	NEI	30 min per time, 1 time per day for 3 months	➀
Li et al. (53)	35/35	25/10 30/5	72.00 ± 2.50/ 73.00 ± 3.00	22.00 ± 7.00/ 21.00 ± 8.00	TJQ	NEI	40 min per time, 1 time per day for 6 months	➀
Li et al. (54)	26/23	19/7 17/6	65.00 ± 2.60/ 66.00 ± 2.80	14.00 ± 2.10/ 13.00 ± 1.50	TJQ	NEI	30 min per time, 5~7 times per week, for 3 months	➀
Wang et al. (55)	26/24	23/3 21/3	67.83 ± 5.32/ 67.86 ± 5.98	7.60 ± 7.75/ 8.84 ± 7.18	TJQ	NEI	60 min per time, 3 times per week, for 3 months	➀➁➂
Niu et al. (56)	20/20	19/1 18/2	59.70 ± 2.76/ 61.30 ± 2.89	N/A	TJQ	NEI	30 min per time, 1 time per day for 6 months	➁
Wei et al. (57)	48/45	41/7 36/9	58.66 ± 7.56/ 58.64 ± 7.52	N/A	WQX	NEI	30 min per time, 1 time per day for 6 months	➀
Jia et al. (58)	26/21	12/14 11/10	53.53 ± 10.05/ 55.46 ± 9.87	N/A	WQX	NEI	45 min per time, 1 time per day for 3 months	➀➁
Liu et al. (59)	50/50	40/10 37/13	74.24 ± 9.10/ 67.72 ± 9.26	N/A	WQX	NEI	45 min per time, 1 time per day for 3 months	➀➁
Gao et al. (60)	36/36	N/A	67.14 ± 9.08/ 66.03 ± 8.18	13.69 ± 5.67/ 14.78 ± 9.24	WQX	NEI	30 min per time, 2 times per day for 3 months	➀➁
Zhao et al. (61)	30/30	20/10 19/11	58.91 ± 5.86/ 56.66 ± 6.43	6.81 ± 2.34/ 6.52 ± 2.42	WQX	NEI	40 min per time, 3 times per week, for 3 months	➀➁
Zhu et al. (62)	26/21	12/14 11/10	53.53 ± 10.05/ 55.46 ± 9.87	N/A	WQX	NEI	45 min per time, 1 time per day for 3 months	➀➁
Cheng et al. (63)	48/45	41/7 36/9	58.66 ± 7.56/ 58.64 ± 7.52	N/A	WQX	NEI	30 min per time, 1~2 times per day for 6 months	➂
Zhang et al. (64)	20/25	13/7 16/9	61.77 ± 4.07/ 59.35 ± 5.27	N/A	YJJ	NEI	60 min per time, 4 times per week, for 6 months	➀➁
Zhang et al. (65)	42/45	33/9 35/10	64.77 ± 11.07/ 62.35 ± 9.27	11.16 ± 2.75/ 10.63 ± 3.37	YJJ	NEI	60 min per time, 1 time per day for 6 months	➀➁➂

*E, experimental group; C, control group; N/A, not available; TJQ, Taijiquan; BDJ, Baduanjin; WQX, Wuqinxi; YJJ, Yijinjing; LZJ, Liuzijue; NEI, non-exercise intervention; ➀, FEV_1_/FVC%; ➁, 6MWT; ➂, CAT*.

### Risk of Bias Assessment

**Figure 2:**
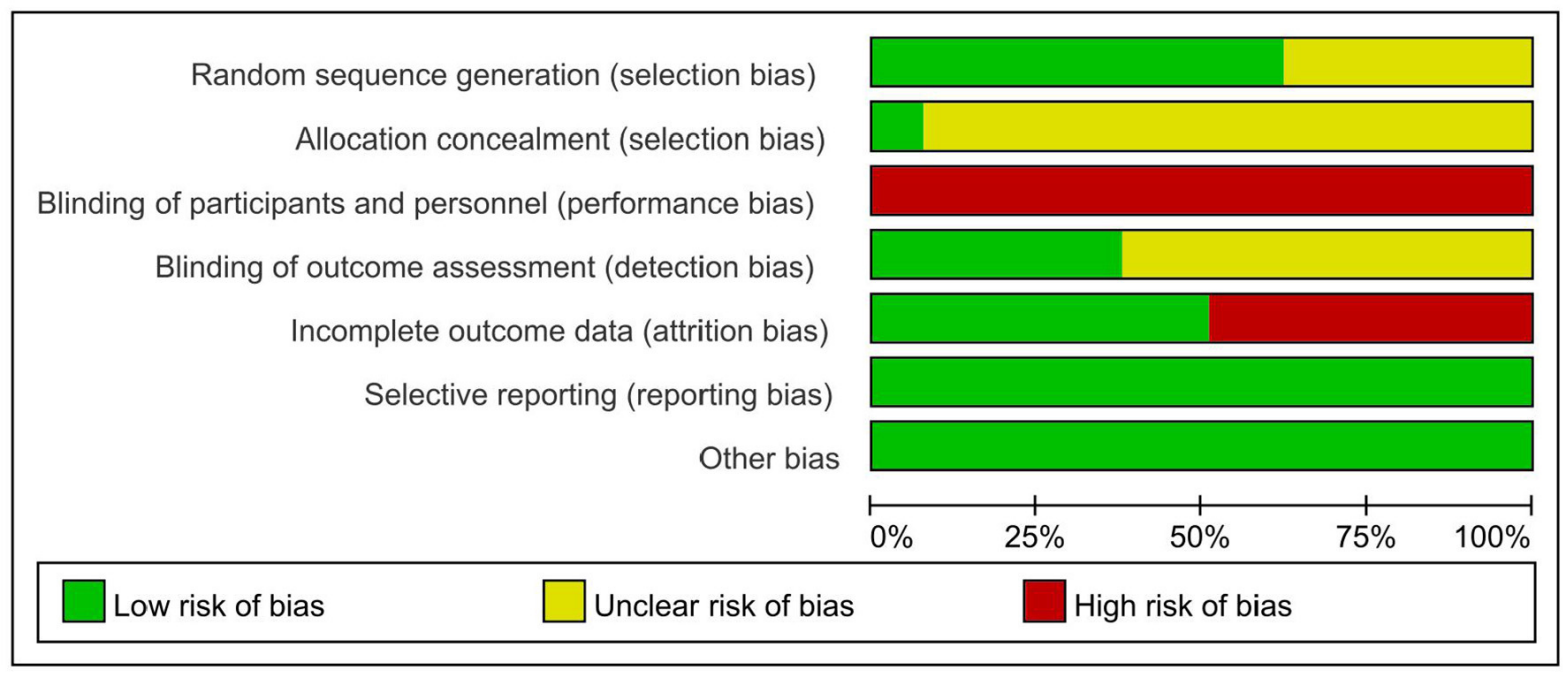
Risk of bias assessment.

Randomization was mentioned in 53 RCTs (13–65), 33 RCTs (13–64) described the specific methods for generating random sequences, and only 4 RCTs (21, 41, 43, 60) were related to allocation concealment. The study itself could not be blinded to researchers or patients, and 20 RCTs (13–65) were blinded to the outcome indicators. Data integrity, selective reporting, and other risks of bias are shown in Figure 2.

### Network Meta-Analysis

**Figure 3:**
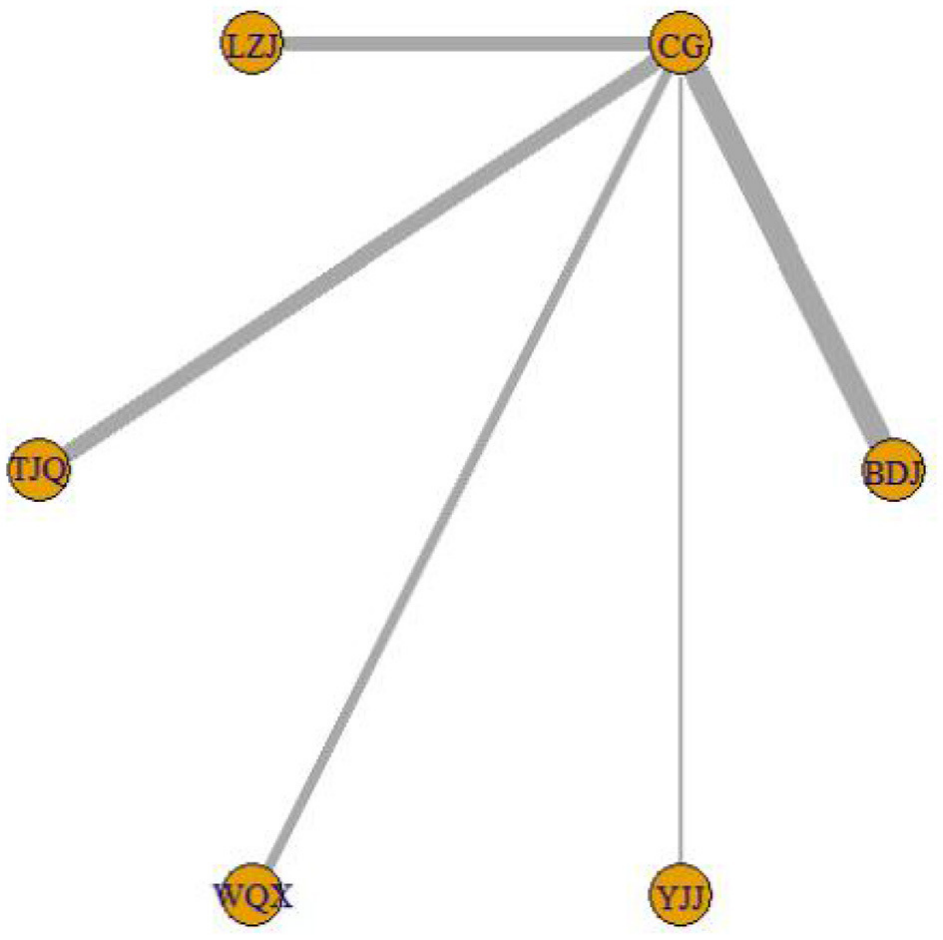
Network diagram of TCEs for patients with COPD. BDJ, Baduanjin Group; LZJ, Liuzijue Group; TJQ, Taijiquan Group; WQX, Wuqinxi Group; YJJ, Yijinjing Group; CG, Control, Group.

**Figure 4:**
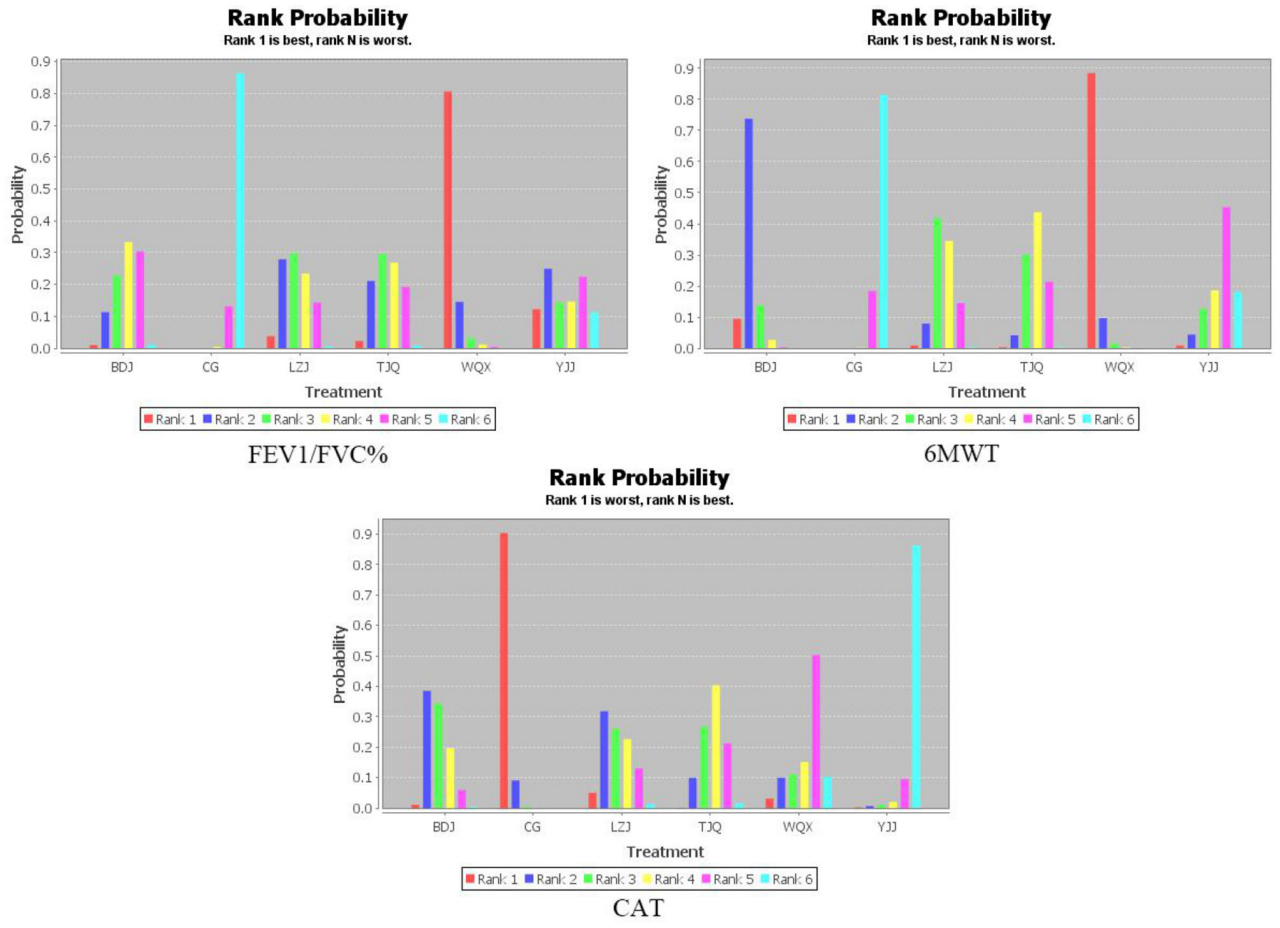
Probability ranking of different interventions and outcome indicators. BDJ, Baduanjin Group; LZJ, Liuzijue Group; TJQ, Taijiquan Group; WQX, Wuqinxi Group; YJJ, Yijinjing Group; CG, Control, Group.

The network diagram of TCEs for patients with COPD is shown in Figure 3. The connection between two yellow balls indicates directly comparable RCTs between the two interventions, and no connection indicates no directly comparable RCTs, which can be indirectly compared using the control group as a reference. The thicker line between the two yellow balls indicates a higher number of RCTs. Consistency analysis of FEV1/FVC%, 6MWT, and CAT outcome indicators from 53 studies showed that all PSRF parameters were close to 1.00, indicating good convergence of network meta-analysis. The results of the network meta-analysis and probability ranking are shown in Tables 2, 3, and Figure 4. The positive scoring outcome indicators (FEV1/FVC% and 6MWT) were ranked with the probability by Rank 1, and higher Rank 1 represents great outcome indicators. The CAT negative scoring outcome indicators were ranked with the probability by Rank N. The higher Rank N represents great outcome indicators.

**Table 2:** The results of network meta-analysis.

**Intervention**		**FEV_**1**_/FVC%**	**6MWT**	**CAT**
BDJ vs	LZJ	−0.97 (−5.33, 3.49)	19.29 (−10.93, 48.29)	0.19 (−3.93, 4.11)
	TJQ	−0.60 (−4.97, 3.83)	22.76 (−5.41, 49.81)	0.88 (−1.93, 4.05)
	WQX	−5.15 (−10.47, −0.03)^*^	−20.79 (−53.80, 11.01)	1.94 (−3.28, 7.13)
	YJJ	−0.64 (−8.30, 6.77)	35.00 (−9.35, 79.87)	5.92 (0.23, 11.20)^*^
	CG	3.45 (0.49, 6.44)^*^	53.47 (36.18, 71.58)^*^	−2.45 (−4.72, −0.39)^*^
LZJ vs	TJQ	0.35 (−4.26, 4.98)	3.51 (−28.65, 35.34)	0.69 (−2.96, 4.89)
	WQX	−4.15 (−9.75, 1.19)	−40.02 (−76.53, −4.10)^*^	1.77 (−4.00, 7.51)
	YJJ	0.30 (−7.46, 7.96)	15.82 (−30.40, 62.82)	5.73 (−0.36, 11.58)
	CG	4.43 (1.12, 7.70)^*^	34.32 (10.44, 58.78)^*^	−2.64 (−6.00, 0.76)
TJQ vs	WQX	−4.58 (−10.16, 0.73)	−43.68 (−78.02, −9.40)^*^	1.09 (−4.50, 5.97)
	YJJ	−0.04 (−7.56, 7.48)	12.10 (−33.21, 59.23)	5.03 (−0.66, 10.10)
	CG	4.05 (0.71, 7.34)^*^	30.69 (10.14, 52.87)^*^	−3.35 (−5.61, −1.52)^*^
WQX vs	YJJ	4.52 (−3.48, 12.55)	55.74 (7.58, 105.11)^*^	3.96 (−3.06, 10.67)
	CG	8.62 (4.46, 13.04)^*^	74.29 (47.67, 102.24)^*^	−4.41 (−9.12, 0.36)
YJJ vs	CG	4.11 (−2.70, 11.14)	18.63 (−22.02, 59.59)	−8.38 (−13.24, −3.28)^*^

* *Represents the difference is statistically significant (P < 0.05). BDJ, Baduanjin Group; LZJ, Liuzijue Group; TJQ, Taijiquan Group; WQX, Wuqinxi Group; YJJ, Yijinjing Group; CG, Control, Group*.

**Table 3:** Best probability ranking.

**Intervention**	**FEV_**1**_/FVC%**	**6MWT**	**CAT**
BDJ	0.01	0.09	0.00
LZJ	0.04	0.01	0.01
TJQ	0.02	0.00	0.02
WQX	0.81	0.88	0.10
YJJ	0.12	0.01	0.86
CG	0.00	0.00	0.00

*BDJ, Baduanjin Group; LZJ, Liuzijue Group; TJQ, Taijiquan Group; WQX, Wuqinxi Group; YJJ, Yijinjing Group; CG, Control, Group*.

#### FEV_1_/FVC%

A total of 43 RCTs (13–65) reported FEV_1_/FVC% scores, involving five TCEs and 3,052 patients. The following comparisons are statistically significant: comparison of BDJ and WQX (MD = −5.15, 95% CI −10.47 to −0.03, *P* < 0.05); comparison of BDJ and the control group (MD = 3.45, 95% CI 0.49 to 6.44, *P* < 0.05); comparison of LZJ and the control group (MD = 4.43, 95% CI 1.12 to 7.70, *P* < 0.05); comparison of TJQ and the control group (MD = 4.05, 95% CI 0.71 to 7.34, *P* < 0.05); comparison of WQX and the control group (MD = 8.62, 95% CI 4.46 to 13.04, *P* < 0.05). The other comparisons were not statistically significant (*P* > 0.05). The probability ranking is: WQX (*P* = 0.81) > YJJ (*P* = 0.12) > LZJ (*P* = 0.04) > TJQ (*P* = 0.02) > BDJ (*P* = 0.01).

#### 6MWT

A total of 35 RCTs (15–65) reported 6MWT scores, involving five TCEs and 2,446 patients. The following comparisons are statistically significant: comparison of BDJ and the control group (MD = 53.47, 95% CI 36.18 to 71.58, *P* < 0.05); comparison of the LZJ and the WQX (MD = −40.02, 95% CI −76.53 to −4.10, *P* < 0.05); comparison of the LZJ and the control group (MD = 34.32, 95% CI 10.44 to 58.78, *P* < 0.05); comparison of TJQ and WQX (MD = −43.68, 95% CI −78.02 to −9.40, *P* < 0.05); comparison of TJQ and the control group (MD = 30.69, 95% CI 10.14 to 52.87, *P* < 0.05); comparison of WQX and YJJ (MD = 55.74, 95% CI 7.58 to 105.11, *P* < 0.05); comparison of WQX and the control group (MD = 74.29, 95% CI 47.67 to 102.24, *P* < 0.05). The other comparisons were not statistically significant (*P* > 0.05). The probability ranking is: WQX (*P* = 0.88) > BDJ (*P* = 0.09) > LZJ (*P* = 0.01) = YJJ (*P* = 0.01) > TJQ (*P* = 0).

#### CAT

A total of 15 RCTs (19, 21, 22, 26, 29, 38, 39, 44, 45, 47–49, 55, 63, 65) reported CAT scores, involving five TCEs and 1,269 patients. The following comparisons are statistically significant: comparison of BDJ and YJJ (MD = 5.92, 95% CI 0.23 to 11.20, *P* < 0.05); comparison of BDJ and the control group (MD = −2.46, 95% CI −4.72 to −0.39, *P* < 0.05); comparison of TJQ and the control group (MD = −3.35, 95% CI −5.61 to −1.52, *P* < 0.05); comparison of YJJ and the control group (MD = −8.38, 95% CI −13.24 to −3.28, *P* < 0.05). The other comparisons were not statistically significant (*P* > 0.05). The probability ranking is: YJJ (*P* = 0.86) > WQX (*P* = 0.10) > TJQ (*P* = 0.02) > LZJ (*P* = 0.01) > BDJ (*P* = 0).

### Adverse Effects

Only one study reported that patients had mild chest tightness and shortness of breath during treatment, which could be relieved after a few minutes of rest (21). Other than that, no adverse effects were reported in other studies.

### Publication Bias

**Figure 5:**
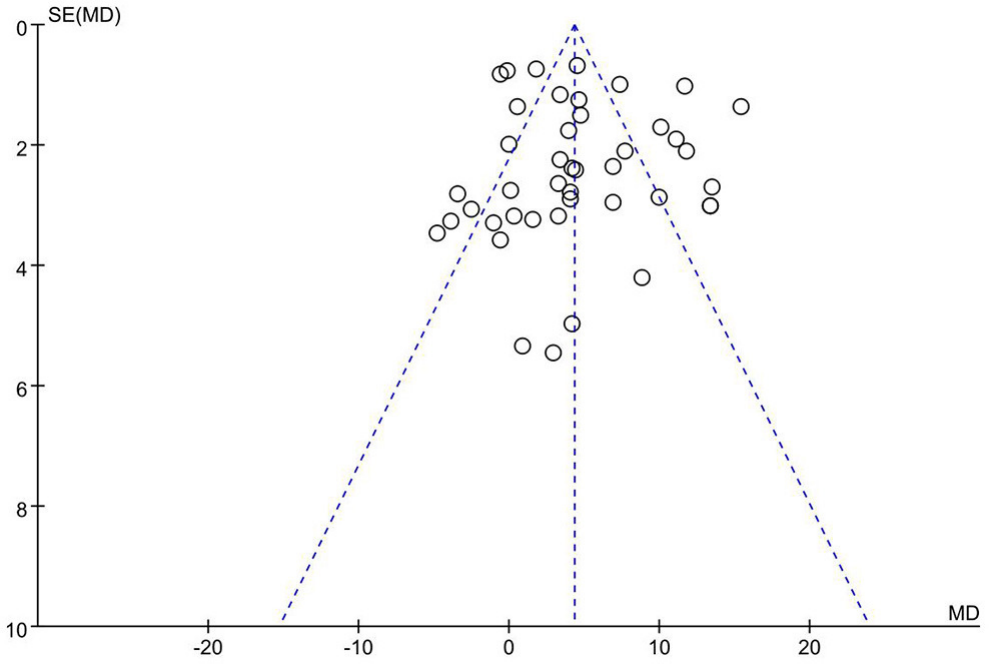
Funnel plot.

Inverted funnel analysis was conducted using FEV_1_/FVC% as the outcome indicator, as shown in Figure 5. The inverted funnel plot is largely symmetrical, indicating no significant publication bias in this study.

## Discussion

Previous studies have shown that each TCE has different effects on COPD patients. Among previous studies, most reported different effects of the same TCE on different outcomes of COPD, but the comparison of different TCEs was not reported. Therefore, which TCE is most effective in alleviating COPD remains unclear.

COPD is one of the most severe public health problems globally. The prevalence rate of COPD in China is 13.7% in people over 40 years old, seriously affecting the quality of life of patients (66). At present, the TCEs promoted by the General Administration of Sport of China mainly include TJQ, BDJ, LZJ, WQX, and YJJ, which are easy to learn, well-accepted, and moderate in intensity. Based on physical, respiratory, and cardiac conditioning, TCEs are gradually gaining more recognition and attention as an effective treatment for COPD through endurance training, respiratory muscle training, stretch exercises, and psychological regulation, which is gradually gaining more recognition and attention (67, 68). In this study, the clinical efficacy of TJQ, BDJ, LZJ, WQX, and YJJ in treating COPD was objectively compared with FEV_1_/FVC%, 6MWT, and CAT scores as outcome indicators, providing a reliable and evidence-based medical basis for clinical treatment.

Shortness of breath and breathing difficulties are two typical symptoms of COPD. In the early stages, these symptoms increase gradually when the patient feels tired, resulting in shortness of breath during daily activities or even at rest (69). Some patients with severe illness or acute exacerbations can have symptoms of wheezing and chest tightness. FEV_1_/FVC% below 70% after the inhalation of bronchodilator is usually used to determine whether airflow is restricted (70). The results showed that WQX has more advantages in improving pulmonary function in patients with COPD. Due to the long duration of COPD, pulmonary function is impaired to varying degrees, resulting in breathing difficulties and restricted mobility. The limitation of activities reduces the adaptability of the body and aggravates the disease. The previous study showed that the decrease in endurance capacity is one of the main characteristics of COPD patients (71). The 6MWT score was used to evaluate the endurance capacity of the patients, with the results showing that WQX was the best intervention with the highest probability. Based on theories and practical guidance of traditional Chinese medicine, WQX incorporates the traditional movement of tigers, deers, bears, apes, and birds, effectively unblocking the meridians, harmonizing the internal organs, preventing diseases, and extending life expectancy. When performing WQX, there is no need to stick to the movement imitation. Trainees should try to embrace the charm of “Five Animals”, discover the majesty of tigers, the calmness of deers, the composure of bears, the dexterity of apes, and the lightness of birds. In this way, trainees can achieve the essentials and spirit of WQX, making them feel comfortable with soft, coordinated, and symmetrical moves (72). These five movements correspond to the five internal organs of the human body. The movements of the bird are beneficial for the lungs. By stretching and lifting upper limbs, breathing can become deeper and more even, and the strength of the respiratory muscles can be enhanced. In addition, this movement can pull the lung meridians and unblock them to a certain extent, thus improving the respiratory function of the lungs (73). However, this network meta-analysis cannot determine the difference in efficacy between the single and compound interventions of WQX, and further studies are required.

As a systemic disease, COPD causes dyspnea and impaired pulmonary function by affecting the lungs. Some systemic effects caused by COPD, such as skeletal muscle dysfunction, can lead to impaired exercise ability, decreased physical activity, and decreased quality of life (74). In addition, patients with moderate or severe COPD can also develop psychiatric symptoms, including anxiety and depression (75). In terms of clinical rehabilitation, the basic goal of the treatment is to increase the self-care of patients, enable their social reintegration, and improve their quality of life. Quality of life is a comprehensive evaluation indicator reflecting the influence of disease status and treatment on physiological function, psychological function, and life of patients (76). Among different evaluation tools, the CAT scale, which is developed based on St George's respiratory disease scale, has certain reliability and validity and is used to evaluate the quality of life of COPD patients (77). The results showed that YJJ has more advantages in improving the pulmonary function of COPD patients. Compared to other TCEs, YJJ emphasizes training in posture, breathing, and mind. The practices of YJJ are in accordance with 12 meridians and the Ren and Du channels of the body. Each move corresponds to the channeling of a merdian (78). Feng (79) showed that practicing YJJ for 24 weeks can increase the number of NK cells, CD4+ cells, and other immune cells. Moreover, the levels of immune factors such as IL-2 and TNF-α can be increased, thus delaying aging and improving the cognitive ability, mobility, and daily living skills in older people.

The American Academy of Sports Medicine recommends that adults should perform aerobic exercises at least 3–5 times a week. However, it is better for adults to perform at least 30–60 min of moderate-intensity exercise, 20–60 min of strenuous exercise, or a combination of moderate-intensity and strenuous exercise every day (80). The optimal exercise prescription for COPD patients is unclear (81), which could be an entry point for future studies.

Pulmonary rehabilitation has many advantages, such as improving quality of life and exercise ability, improving psychological status, and reducing morbidity and mortality. However, studies have shown that <10% of COPD patients have access to specialist services (82, 83). This phenomenon is caused by many reasons, such as the poor medical environment in rural areas, mobilities problems of patients, and family dependency. All these problems reduce the accessibility and feasibility of pulmonary rehabilitation. Therefore, it is essential to find alternative strategies for pulmonary rehabilitation. TCEs, such as WQX and YJJ, are not limited by time and space, offering alternative methods to improve the accessibility to pulmonary rehabilitation. This result is consistent with the policy statement of the American Thoracic Society/European Respiratory Society on pulmonary rehabilitation, which states that increasing the accessibility to pulmonary rehabilitation is a key priority (84). However, most of the current studies are limited to the effects of a single TCE. Therefore, the efficacy and mechanism of different therapies should be investigated in future studies.

This network meta-analysis still has some potential bias. The 53 included RCTs (12–65) were all published in Chinese and English, which might be selectivity biased. In addition, only four RCTs (28, 48, 59, 64) employed sealed envelopes for concealed grouping, and there were no restrictions on blind methods of included studies. Moreover, the severity of the disease and the subjectivity of the outcome indicators could affect the results of the network meta-analysis. Therefore, more large-scale, double-blind, and high-quality RCTs are needed to verify the findings in this study.

## Conclusions

In conclusion, the existing evidence shows that WQX has more advantages in improving pulmonary function and endurance capacity in COPD patients, while YJJ is better in improving quality of life. In addition, TCE has no significant adverse effects in treating COPD, which is recommended to be applied and promoted in clinical practices.

## Data Availability Statement

The original contributions presented in the study are included in the article/supplementary material, further inquiries can be directed to the corresponding author.

## Author Contributions

LL designed and wrote this study. HH provided guidance regarding the methodology. YY and JS reviewed the full manuscript. YW and XD took part in the data selection and extraction. YJ and LH performed the statistical analysis and analyzed the data. All authors contributed to the article and approved the submitted version.

## Funding

This study was funded by Shandong Traditional Chinese Medicine Science and Technology Development Planning (No. 2017-018), Shandong University of Traditional Chinese Medicine Research and Innovation Outstanding Team (No. 220316), and Shandong Provincial Universities Scientific Research Development Planning (No. J18KB130).

## Conflict of Interest

The authors declare that the research was conducted in the absence of any commercial or financial relationships that could be construed as a potential conflict of interest.

## Publisher's Note

All claims expressed in this article are solely those of the authors and do not necessarily represent those of their affiliated organizations, or those of the publisher, the editors and the reviewers. Any product that may be evaluated in this article, or claim that may be made by its manufacturer, is not guaranteed or endorsed by the publisher.
